# Where a Certified Case May Be Treated

**Published:** 1909-04-03

**Authors:** 


### Mental Diseases.

### WHERE A CERTIFIED CASE MAY BE TREATED.

When certified an insane person becomes either a pauper patient or a private patient. In Section 341 of the Lunacy Act of 1890, the definition of a pauper is given as "a person wholly or partly chargeable to a union, county or borough"; and of a private patient as "a patient who is not a pauper." By Section 18 of the same Act a justice may not sign an order for the reception into an institution of a person as a pauper lunatic unless he is satisfied that the alleged pauper is either in receipt of relief, or in such circumstances as to require relief for his proper care: it being added that, for the purposes of this section, a person is to be deemed to be in receipt of relief who is visited by a medical officer of the union at the expense of the union. A certified private patient may at any time be transferred to the pauper class, should he fall under the definition of that class, and similarly a pauper patient may be transferred to the private class, should the required weekly payments be forthcoming.

For a person to be certified as a private patient, a petition from a relative or friend, with a statement of particulars attached, two medical certificates, and a reception order signed by a judicial authority are required. For a pauper patient no petition is required and generally only one medical certificate. The judicial authority to sign the order must be a judge of county courts, a stipendary magistrate, or a justice of the peace specially appointed under the Lunacy Act of 1890. (A list of the justices specially appointed under the Act can be seen by applying to the police, the clerk of the peace, or the clerk to the justices). The steps to be taken for the certification and removal of a patient will depend on which class (private or pauper) he belongs to and where it is proposed to send him to.

A certified patient may be looked after at home or be sent for care and treatment either (1) to single care, or (2) to an institution. By "single care" is meant residence with someone who only looks after the one patient. Any person may take in one certified case to reside with them "for profit" provided that certain relations set forth in the Act do not exist between the patient and themselves—the conditions as to reports, visiting, etc., also set forth in the Act have of course to be fulfilled. More than one case may not be taken by any person without the special consent of the Commissioners in Lunacy.

Institutions to which the certified insane may be sent are of three kinds—namely (1) licensed houses (or private asylums), (2) registered hospitals, and (3) county and borough asylums. These correspond more or less with the three kinds of institutions in which cases of physical illness may be treated, that is, (*a*) private hospitals or nursing homes, (*b*) general hospitals, and (*c*) poor-law infirmaries. Licensed houses are private asylums, licensed by the authorities under the Lunacy Act for the reception of a specified number of patients. They are the private business of those to whom the license is granted, and the fees charged are fixed by the license holders. No accounts are published, but they are, of course, strictly under the control of the Commissioners in Lunacy as regards the treatment of patients. A list of these licensed houses is to be found in the annual report of the Lunacy Commission, together with the names of the licensees and medical superintendent, and the number of patients of each sex for which the license is granted. The fees charged at any of them may be ascertained by application to the resident medical superintendent.

The registered hospitals for those afflicted with mental diseases differ from most of the general hospitals for physical diseases, in that payment towards maintenance is taken from the friends of any patient who can afford it. They are institutions, "not being asylums," registered under the Lunacy Act by the Commissioners "wherein lunatics are received and supported wholly or partly by voluntary contributions, or by any charitable bequest or gift, or by applying the excess of payments of some patients for or towards the support, provision, or benefit of other patients." They are managed by committees of subscribers, and are obliged by the Act to publish annually a full statement of accounts in the form prescribed by the Commissioners. In most of them certain kinds of cases are ineligible—*e.g.* epileptics, paupers, etc., and only acute recoverable cases are taken free or at low fees. The fees paid in any case are arranged between the committee and friends of the patient. A list of the registered hospitals will be found in the annual report of the Commission, and application for admission of patients should be made to the Secretary or medical superintendent.

Neither licensed houses nor registered hospitals are under any obligation to take in any particular case of mental disorder; nor, unless some definite agreement has been previously made, to keep the patient for any definite length of time after admission. Thus, unless arrangements are made beforehand, it is useless taking the patient, even if properly certified, to the institution, as admission can be refused: and if, after admission, the case is found to be ineligible, the friends can be called upon to remove it.

